# PRISM: Fighting Against Open Access

**DOI:** 10.3332/ecancer.2007.ed2

**Published:** 2007-09-21

**Authors:** Linda Cairns

**Affiliations:** European Institute of Oncology, Milan, Italy

Open Access (OA), made possible by the advent of the internet, is immediate, free and unrestricted online access to scholarly material; primarily peer-reviewed research articles in journals.

This can be read, downloaded, copied, distributed, and used (with attribution) in any way.

Promoted by groups such as Public Library of Science (PLOS) and the Max Planck Institute, the debate over its benefits has also recently been taken up by PRISM, the Partnership for Research Integrity in Science & Medicine. It is a group of scientific and medical activists who are fighting against OA for research articles. They claim that by keeping research closed to the public they can “preserve the integrity of America’s scholarly research”.

A visit to the website http://www.prismcoalition.org/ reveals the true nature of their argument. The site amounts to an anti-OA forum, aimed at counteracting the accelerating growth of OA. The people behind PRISM, the Association of American Publishers (AAP) originally stated that they are against OA because it:

- “undermines the peer review process.”
- “opens the door to scientific censorship”. (They failed to explain how making research publications freely accessible will lead to censorship)
- “undermines the reasonable protections of copyright holders.”

Not all the American publishing companies were in agreement, however. Mike Rossner, Executive Director of Rockefeller University Press, wrote to the AAP stating that they strongly disagree with the views presented on the PRISM website. His views were echoed by the Association of Research Libraries and many commentators online.

The backlash surrounding PRISM’s original statement resulted in an update of the site, with a more measured tone on the front page, including the deletion of the statement regarding scientific censorship. There is still no mention of those members of the AAP who disagreed with PRISM.

PRISM’s ‘pit-bull’ PR tactics are unlikely to win them any friends. In taking such an aggressive stance they may well alienate those who have agreed, at least in part, with their criticisms. Is it the last gasp from publishers who refuse to do what is the right thing for science and society? (I.e. open up their publications to those who paid for the original research, namely the public).

Unlike the pro-OA lobby, which has a huge and growing public support base worldwide, the anti-OA lobby has neither public support, nor any ethical or practical case with which to build it on.

The arrival of *e*cancermedicalscience has not exactly been welcomed with open arms by print publishers, but then, it’s not-for-profit, and science publishing stands for very substantial profit.

